# Supplementary material for: What Is the Best Treatment Choice for Concomitant Ipsilateral Femoral Neck and Intertrochanteric Fracture? A Retrospective Comparative Analysis of 115 Consecutive Patients
Source: J Pers Med. 2022 Nov 16;12(11):1908. doi: 10.3390/jpm12111908 (PMC9693868; doi:10.3390/jpm12111908)
Supplement: Supplementary file 1 [file jpm-12-01908-s001.zip › jpm-1988914-supplementary.pdf]

Supplementary Table S1

Table S1. Summary of literatures regarding concomitant ipsilateral femoral neck and intertrochanter fracture.

**Author (year)**	**Study design & period**	**Cases (Incidence)**	**Age**	**Sex F:M**	**Mechanism**	**Fracture type**	**Diagnosis**	**Operation**	**F/U (month)**	**Complication**
**An (1989) [4]**	Case/NS	1	97	0:1	LE	Type II	Intraop	HR	NS	None
**Pemberton (1989) [17]**	Case/NS	1	73	1:0	LE	Type II	Xry	DHS	30	None
**Isaacs (1993) [9]**	Case/NS	1	72	1:0	LE	Type I	CT	Screw	NA	Death*
**Taylor (1996) [25]**	Case/NS	1	89	1:0	LE	Type I	Xry	DHS	1	Death*
**Taylor (1996) [25]**	Case/NS	1	74	0:1	LE	Type II	Xry	DHS	108	Nonunion
**Cohen (1999) [7]**	Case/NS	1	79	1:0	LE	Type II	Intraop	DHS	24	None
**Kumar (2001) [12]**	Case/NS	1	83	1:0	LE	Type II	Xry	DHS	12	ONFH
**Kyle (2005) [13]**	Retrospective /10 year	20 (8%)	63.6	7:13	16LE:4HE	Type II	Xry	DHS	17	3 nonunion, 5 mechanical, 1 ONFH
**Lakshmanan (2005) [14]**	Case/NS	1	91	1:0	LE	Type III	Xry	HR	6	None
**Sayegh (2005) [21]**	Case/NS	1	54	0:1	HE	Type II	Xry	DHS	58	LLD

**Butt (2007) [6]**	Case/NS	1	30	0:1	HE	Type II	Xry	DCS	12	None
**Poulter (2007) [19]**	Case/NS	1	76	1:0	LE	Type I	Xry	Plate	4	None
**Dhar (2008) [8]**	Case/2006	1	30	0:1	HE	Type II	Xry	Plate	12	None
**Perry (2008) [18]**	Case/NS	1	86	1:0	LE	Type I	Postop Xry	DHS	12	ONFH
**Loupasis (2010) [15]**	Case/NS	1	36	0:1	HE	Type II	X-ray	DHS	24	None
**Neogi (2011) [16]**	Case/NS	1	28	0:1	HE	Type II	CT	DCS	28	None
**Taniguchi (2013) [24]**	Case/NS	1	76	0:1	LE	Type II	CT	HR	6	None
**Tahir (2014) [23]**	Case/NS	1	87	1:0	LE	Type II	CT	HR	17	None
**Khan (2017) [10]**	Case/NS	1	66	0:1	LE	Type II	Xry	HR	18	None
**Saleeb (2017) [20]**	Case/NS	1	88	1:0	LE	Type I	CT	HR	NS	Dislocation
**Yoo (2017) [29]**	Retrospective /2001-2014	33 (9%)	76.25	29:4	LE	Type II	CT	CMN	21.8	4 mechanical 1 nonunion
**Videla (2017) [27]**	Retrospective /2010-2016	31 (1.3%)	81.5	24:9	LE	Type II	CT	HR4 DHS 13 CMN 12	NS	None
**Kothari (2019) [11]**	Case/NS	1	72	1:0	LE	Type II	CT	HR	3	None
**Ahmed (2020) [3]**	Case/NS	1	26	1:0	HE	Type II	CT	Plate	60	None
**Su (2020) [22]**	Case/NS	1	38	1:0	HE	Type II	CT	Plate	84	ONFH

**Su (2020) [22]**	Case/NS	1	29	0:1	HE	Type II	CT	Plate	36	None
**Biesmans (2021) [5]**	Case/NS	1	42	0:1	HE	Type II	CT	HR	12	None
**Tong (2021) [26]**	Retrospective /2012-2018	19	61.3	9:10	8LE:11HE	Type II	CT	Screw 1<br>CMN 8<br>DHS 4<br>Plate 5<br>HR 1	13	1 nonunion, 2 mechanical
**Vluggen (2021) [28]**	Case/NS	1	82	1:0	LE	Type II	CT	HR	11	none

The literatures are listed in chronological order, and any cases from studies, in which the total number of cases are below 10, are listed individually on separate rows.
*Death: death by unrelated cause; HE: high energy; LE: low energy; STC: subtrochanteric fracture; ITC: intertrochanteric fracture; SC: subcapital femoral neck; TC: transcapital femoral neck; Xry: X-ray; CT: computed tomography; CMN: cephalomedullary nai; HR: hip replacement; DCS: dynamic condylar screw; DHS: dynamic hip screw; NS: not specified; ONFH: osteonecrosis of the femoral head; LLD: limb length discrepancy
